# A case report of ectopic pancreatitis in an isolated enteric duplication cyst

**DOI:** 10.1186/s12893-019-0531-7

**Published:** 2019-06-18

**Authors:** Yoshikazu Tanaka, Go Nakai, Hideki Tomiyama, Yoshitaka Kurisu, Yoshifumi Narumi

**Affiliations:** 1 Department of Radiology, Osaka Medical College, 2-7 Daigakumachi, Takatsuki, Osaka, Japan; 2 Department of Surgery, Osaka Medical College, 2-7 Daigakumachi, Takatsuki, Osaka, Japan; 3 Department of Pathology, Osaka Medical College, 2-7 Daigakumachi, Takatsuki, Osaka, Japan

**Keywords:** Isolated enteric duplication cyst, Mesenteric Meckel’s diverticulum, Ectopic pancreas

## Abstract

**Background:**

Isolated enteric duplication cyst is an intestinal duplication cyst found in a distant location from the intestinal tract and it is said to have its own blood supply. Meckel’s diverticulm is considered as an antimesenteric structure and has its own blood supply. However, there are some reported cases of Meckele’s diverticum in the mesenteric side. Ectopic pancreas may be found in both entities.

**Case presentation:**

A 5-year-old girl presented with increasing abdominal pain around the umbilicus. On laboratory investigation serum pancreatic enzymes and C-reactive protein were elevated. Abdominal computed tomography (CT) revealed a normal pancreas but a cystic lesion in the mesentery of the ileum. A nodule with a marked enhancement was observed in the wall of the lesion. During the laparoscopy, the lesion was found at the root of the mesentery and was distant from the ileum. The lesion was resected suspecting an abscess. Pathologically, the wall of the lesion consisted of small bowel like tissue, and pancreatic tissue was seen beneath the mucosa. There were some post inflammatory changes in the pancreatic tissue. Retrospectively on thin slice enhanced CT, an independent blood supply was noted. Based on these findings, a diagnosis of ectopic pancreatitis in an iliac intestinal duplication cyst was made.

**Conclusion:**

Isolated enteric duplication cyst in the root of ileal mesentery and mesenteric Meckel’s diverticulum have similarities. In the present case, the diagnosis of isolated enteric duplication cyst was made since it was found distant from the ileum. It is important to consider the possibility of ectopic pancreatitis when serum pancreatic enzymes are elevated even when the pancreas appears normal.

## Background

Enteric duplication is a rare anomaly, which is found along the gastrointestinal tract and generally shares the same blood supply as the nearby intestine [1]. It has been reported to contain ectopic pancreatic tissue in approximately 3% of cases [2]. Isolated enteric duplication cyst is an intestinal duplication cyst occurring in a location independent of the gastrointestinal tract and it has its own blood supply [3].

## Case presentation

A 5-year-old girl with no past medical or surgical history had complained of waxing and waning pain around the umbilicus for a few years. On several occasions, she had visited her general practitioner due to abdominal pain, but no abnormalities had been noted. The pain recurred with greater severity, and abdominal ultrasound performed by her general practitioner revealed a cystic lesion in the right lower abdominal cavity. An abdominal abscess was suspected thus she was referred to a tertiary hospital.

**Fig. 1:**
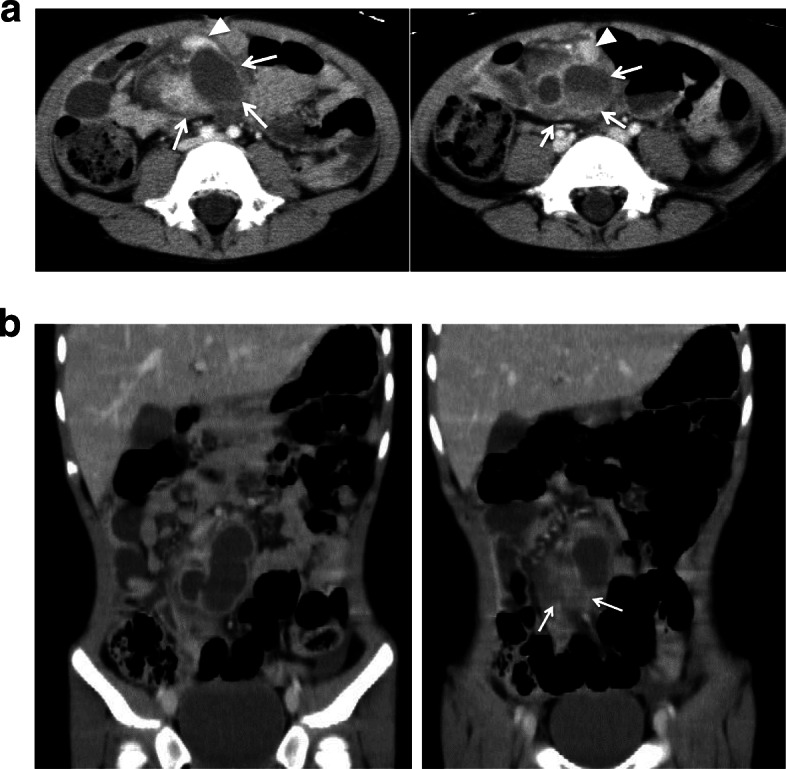
Enhanced CT. **a** In the ileal mesentery at the level of the umbilicus, a cystic lesion with enhancing wall is seen (arrows). There is a enhancing nodule in the cystic wall (arrow heads). **b** On coronal plane, the lesion appears to be a tubular structure. The fat density around the lesion is increased suggesting inflammation (arrows)

**Fig. 2:**
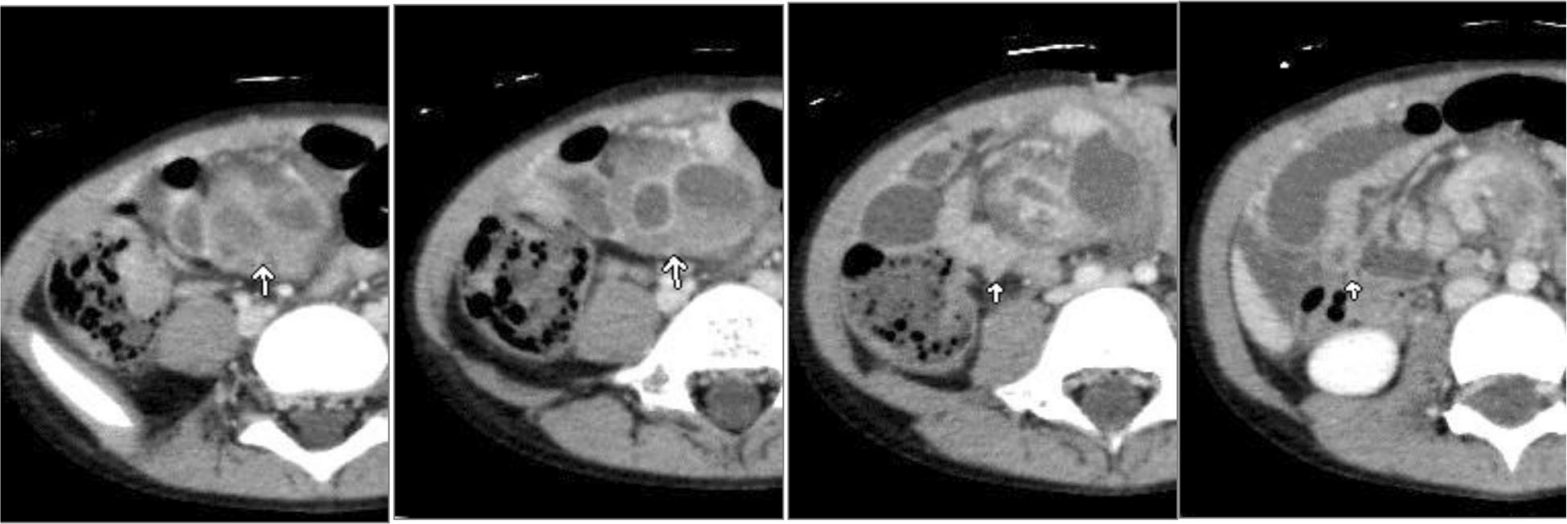
Enhanced CT, retrospective finding. An independent blood vessel coursing through the dorsal side into the lesion was identified (arrows)

On admission, the vital signs were as follows: blood pressure, 125/81 mmHg; pulse, 166 beats per minute; and temperature; 36.5 °C. On palpation, rebound tenderness was noted on the right iliac fossa. On laboratory investigation, the white blood cell count was 17,400/μL and the C-reactive protein level was 1.54 mg/dl. There was an elevation in the serum pancreatic amylase and lipase level (124 U/L, 114 U/L respectively). Contrast enhanced abdominopelvic computed tomography (CT) (Fig. 1) revealed a cystic lesion measuring 6 × 5 cm with an enhancing thick wall in the ileal mesentery. The cystic lesion was tubular in shape and inflammation was suspected as the density of the adjacent fat was increased. A small enhancing nodule in the cyst wall was also noted. Retrospectively, a distinct blood vessel coursing on the dorsal side into the lesion was identified (Fig. 2). The pancreas appeared within normal limits. The appendix was slightly enlarged and a diagnosis of appendicitis was equivocal.

**Fig. 3:**
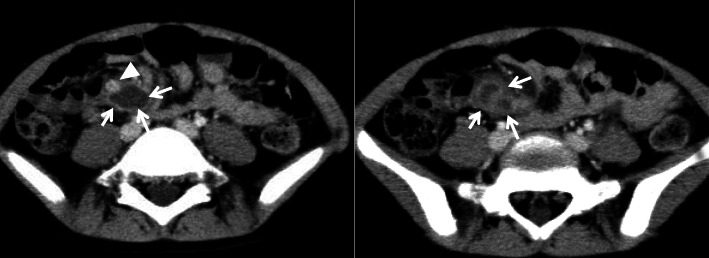
Follow-up CT. On the follow up CT at 3 months revealed, decrease in size of the lesion is seen. There is a enhancing nodule seen in the cystic wall (arrow)

An abscess in the mesentery was suspected, and the patient was put on nil by mouth and treated with antibiotics. There were significant improvements in the symptoms and the laboratory data on the second week and she was discharged on the fifth week. Follow-up enhanced CT at 3 months showed a significant decrease in size of the cystic mass (Fig. 3). However, the abdominal pain persisted and the possibility of appendicitis with abscess formation was considered, hence laparoscopic appendectomy was performed. After appendectomy, the abdominal pain recurred and a slight elevation of the pancreatic enzymes persisted so a laparoscopy was repeated for possible resection of the mesenteric lesion. During laparoscopy, a cystic mass was found in the root of the iliac mesentery measuring approximately 2 × 1.5 cm in diameter. There was no communication between the mass and the nearby ileum. During the procedure, there was bleeding from the blood vessel coursing dorsal to the mass and hemostasis was required.

**Fig. 4:**
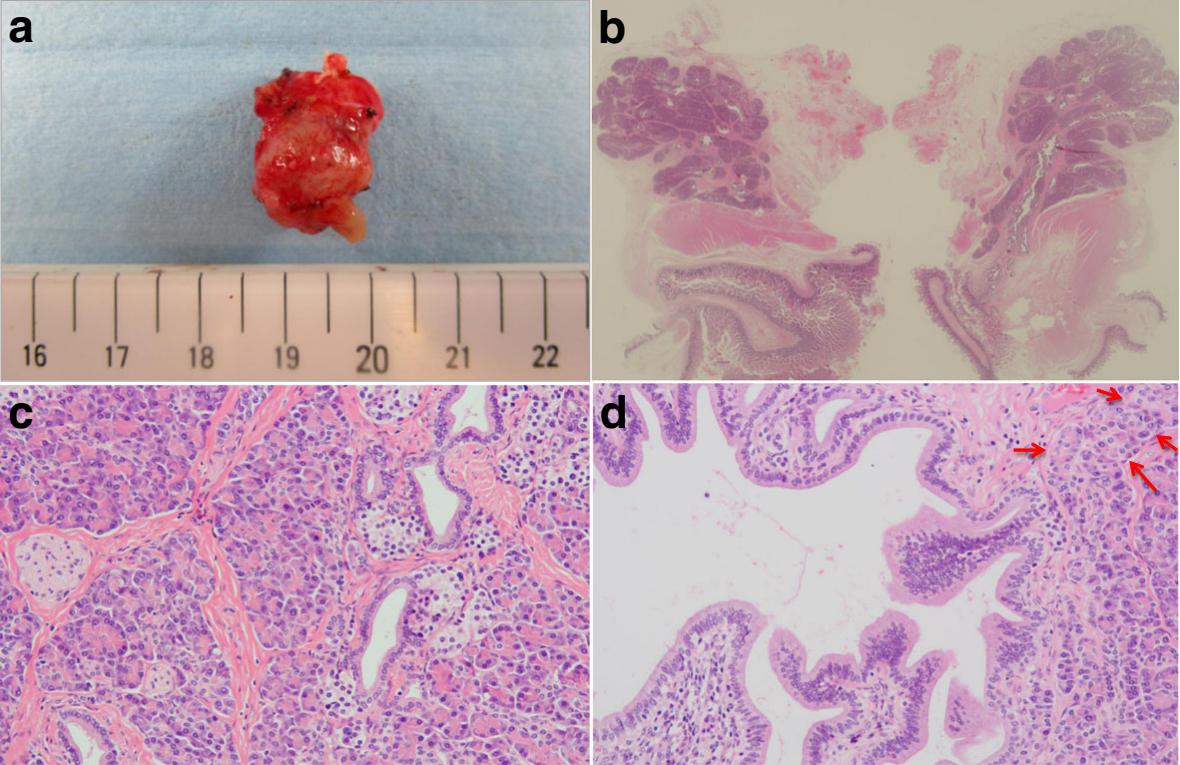
Pathological findings. **a** Gross appearance of the resected lesion. **b** On loupe image, the cystic wall was composed of small intestine wall with villi in the mucosa. Ectopic pancreatic lobules are seen in the cystic wall. **c** On high power view, acini, islets of Langerhans and pancreatic ducts are seen in the pancreatic tissue. **d** Lymphocytes infiltration and acinar loss with islet-cell pseudohyperplasia (arrows), which are evidence of post pancreatitis, are observed

Pathologically, the wall of the lesion assimilated the small bowel wall with villi in the mucosa and under the mucosa there was pancreatic tissue consisted of acini, ducts and islets of Langerhans (Fig. 4). The pancreatic duct appeared to open into the cyst. Therefore, a diagnosis of isolated enteric duplication cyst with Heinrich type I ectopic pancreas was made. Changes suggestive of post pancreatitis such as lymphocytes infiltration and acinar loss with islet-cell pseudohyperplasia were also seen in the pancreatic tissue. Postoperatively there were no complications and in the follow-up the serum pancreatic enzymes normalized. The patient reported no recurrence of abdominal pain since the resection of the lesion.

## Discussion and Conclusion

Enteric duplication is a rare gastrointestinal anomaly with an incidence of 2 in 9000 among postmortem newborn infants and fetuses [4]. It can occur anywhere along the intestinal tract, although about 44% of duplication cysts are found in the small intestine [5] and most cases are seen in the ileum. It is defined as a structure consisting of intestinal mucosa surrounded by smooth muscle, and it shares the same smooth muscle and blood supply with the nearby intestine [1]. It has been reported that ectopic gastric mucosa is observed in 20–30% of cases of enteric duplication [6–8], while ectopic pancreas is found in 3% of cases [2].

Some cases of enteric duplication are not anatomically associated with the gastrointestinal tract and are designated as isolated enteric duplication cysts [3]. In contrast to enteric duplication, isolated enteric duplication cysts have their own blood supply. There have been several reported cases of isolated enteric duplication cysts including the tongue, pleural space, liver and pancreas, biliary tree, and retroperitoneum. However, the number of reports is still limited. Another possibility for the diagnosis was mesenteric Meckel’s diverticulum. Meckel’s diverticulum is a remnant of the embryonic vitelline duct which is a tubular structure connecting the yolk sac and the embryonic midgut during the embryonic development. It is obliterated at the 6th – 7th week, but in Meckel’s diverticulum, it fails to obliterate and remains as a diverticulum [9]. Meckel’s diverticulum is the most frequently found anomaly in the gastrointestinal tract, and is found in around 2–3% of the population [10]. Anatomically, Meckel’s diverticulum is found within 100 cm from the ileocecal valve at the antimesenteric side [11, 12] and its wall consists of normal small bowel wall with five layers and it has its own blood supply through the mesodiverticular band. Ectopic gastric mucosa is found in approximately 23–50% of cases [13, 14], while ectopic pancreatic tissue is found in about 5–16% of cases [15]. Ectopic pancreas is most frequently found in the gastrointestinal tract around the pancreas, such as the stomach and duodenum. Meckel’s diverticulum accounts about 5% of all ectopic pancreas cases. Interestingly ectopic pancreas is also found outside the gastrointestinal tract and these include the gallbladder, spleen, lungs and umbilicus [16]. Although one of the cardinal features of Meckel’s diverticulum is its antimesenteric location, there have been a few reported cases of mesenteric Meckel’s diverticulum [17–21]. These reported cases are either ileal diverticulum or a cystic mass with the wall consisted of small intestine in the mesenteric side. However, especially in cystic cases without a communication with the ileum, the diagnosis seems ambiguous.

In the present case, the wall of the cystic lesion consisted of small bowel wall without sharing the smooth muscle with the nearby ileum and it had its own blood supply. Nonetheless its location was at a feasible distance from the ilioceolovalve for Meckel’s diverticulum and pancreatic tissue is observed more frequently in Meckel’s diverticulum, it was distant from the small intestine siting at the root of the mesentery almost in the retroperitoneum. These findings support the diagnosis of an isolated enteric duplication cyst.

We have experienced a case of ectopic pancreatitis in an isolated intestinal duplication. It had a distinguished blood supply and Mesenteric Meckel’s diverticulum was considered for the differential diagnosis. It was located at the root of the mesentery where the lesion was distant from the border of the ileum, thus the diagnosis of ectopic pancreatitis in an isolated intestinal duplication was made. It is important to consider ectopic pancreatitis when serum pancreatic enzymes are elevated even when the pancreas appears normal.

## Data Availability

The datasets used and analyzed during the current study are available from the corresponding author on reasonable request.

## Abbreviation

CT: Computed tomography
